# Hazards and health problems in occupations dominated by aged workers in South Korea

**DOI:** 10.1186/s40557-017-0177-9

**Published:** 2017-06-26

**Authors:** Jungsun Park, Soo Geun Kim, Jong-shik Park, Boyoung Han, Kab Bae Kim, Yangho Kim

**Affiliations:** 1 Department of Occupational Health, Catholic University of Daegu, Gyeongsan, South Korea; 2 Department of Occupational Medicine, Kangbuk Samsung Hospital, Sungkyunkwan University, School of Medicine, Seoul, South Korea; 3 Yonsei University, Seoul, South Korea; 4 Occupational Safety and Health Research Institute, Korea Occupational Safety and Health Agency, Ulsan, South Korea; 5 Department of Occupational and Environmental Medicine, Ulsan University Hospital, University of Ulsan College of Medicine, # 290-3 Cheonha-dong, Dong-gu, Ulsan, 682-060 South Korea

**Keywords:** Hazards, Health problem, Aged, Occupation, Musculoskeletal disorders

## Abstract

**Background:**

South Korea’s population is aging more rapidly than any other country. Aging of the productive population will lead to shortage of labor and the decreasing quality of the labor force in South Korea. South Korea needs health care strategies to support the establishment of work environments that are appropriate for elderly workers who have reduced physical capacity. This paper aims to identify occupations that are dominated by aged workers and assess the exposure to hazards and work-related health problems of aged workers in these occupations.

**Methods:**

We identified the 20 occupations in South Korea that employ the most aged workers (at least 55 years-old), among all 149 occupations that are defined as minor categories (identified by three digits) by the Korean Standard Classification of Occupations (KSCO). Exposure to hazards and work-related health problems of individuals in these occupations were evaluated by analyzing the results of the fourth Working Conditions Survey of 2014.

**Results:**

Among the 20 occupations that employ the most aged workers, ‘Elementary Occupations’, which the KSCO classifies as major category (9), had the largest proportion of aged workers. After this, there were five occupations of skilled manual workers and six occupations of skilled non-manual workers. Aged workers in elementary and skilled manual occupations reported frequent exposure to job-specific hazards, such as noise, vibrations, high and low temperatures, solvents, and chemicals. Relative to other workers, aged workers in the occupations reported more frequent exposure to ergonomic hazards, such as tiring or painful positions, carrying or moving heavy loads, and repetitive movements, and also reported more work-related musculoskeletal disorders and general fatigue. Injury due to accident was common in machinery-handling occupations.

**Conclusion:**

Job-specific hazards should be reduced to prevent occupation-related disorders in elementary and skilled manual occupations that are dominated by aged workers.

## Background

South Korea’s population is aging more rapidly than any other country [1, 2]. It became an aging society in 2000, and will be an aged society by 2018 and a super-aged society by 2026 [1]. Labor shortages and a lack of skilled workers are expected due to reductions in the size of the productive population (15–64 years-old) in 2017 and the mass retirement of “baby boomers”, who were born after the Korean War. Further, the most economically active age group (30–49 years-old) will decline continuously, from 49% in 2005, to 42% in 2020, and to 37% in 2050. Thus, aging of the productive population will reach the point when most current baby boomers have left the labor market, probably by 2030 [1]. Similarly, the percentage of elderly people (at least 50 years-old) was 25% in 2010, and is expected to exceed 33% in 2020 [1, 3]. The aging of the workforce will likely increase the rate of workplace accidents, because more elderly workers than young workers have degradations of physiological functions, such as sensory systems, sense of equilibrium, and motor control [4, 5]. The increased prevalence of chronic and degenerative diseases due to aging will also lead to a decreased quality of the labor force [6]. Health care strategies that seek to maintain and promote the work ability of elderly employees are needed to meet the impending shortage of labor and the decreasing quality of the labor force in South Korea.

Thus, South Korea needs long-term and short-term strategies that maintain and promote the work ability of aged individuals. One long-term strategy is to prevent non-communicable diseases (NCDs) through workplace health promotion activities, while workers are still actively employed. These activities require the collaboration of governmental agencies at the central and local levels. First, the common goal of central governmental agencies should be reinforcement of follow-up measures in general medical examinations and promotion of healthy lifestyles for workers. Second, collaboration at the local level should be performed at the Worker’s Health Center, the Health Promotion Center, and community health centers [7]. One short-term strategy is to support the establishment of work environments that are appropriate for elderly workers who have reduced physical capacity. Long- and short-term strategies should focus on workplaces that are dominated by aged workers, rather than the entire population of aged workers, because the work ability of aged workers can be efficiently and effectively maintained and promoted when employers take the responsibility to lead such activities, and workers will then be more likely to participate. Thus, we must first identify the occupations that employ the most aged workers, and the prevalence of different occupational hazards at these workplaces. Second, we must improve the health and safety of the elderly workers in their workplaces by developing and disseminating age-friendly occupational health and safety guidelines, based on job-specific information.

As a short-term strategy, the present study aims to identify occupations that are dominated by aged workers, and assess the exposure to hazards and work-related health problems of aged workers in these occupations.

## Methods

### Materials and subjects

We defined aged workers as those who were at least 55 years-old, according to Enforcement Decree of the Act on Prohibition of Age Discrimination in Employment and Aged Employment Promotion in Korea.

### Selection of occupations

Study subjects were aged workers and young workers in occupations that were dominated by aged workers, based on raw data of the 2014 Employment Survey by Area (A type) [8]. We initially identified 20 of 149 occupations, in descending order of the number of aged workers. These are minor categories (identified by three digits), as defined by the Korean Standard Classification of Occupations (KSCO) [9].

### Source of data on hazards and health problems

We analyzed the results of the fourth Working Conditions Survey of 2014 by the Korea Occupational Safety and Health Agency to evaluate the characteristics of working conditions, exposure to hazards, and work-related health problems in these 20 occupations. The survey population was a representative sample of those who were aged 15 years or older. Individuals who were retired, unemployed, housewives, or students were excluded. The basic design was a multi-stage random sampling of the enumeration districts used for the 2010 population and housing census. A total of 50,007 interviews were conducted, but we only included the responses of 30,751 employees who had worked in their current workplaces for more than 1 year prior to the survey. Among the 6502 aged workers (55 or older), there were 3482 males (53.6%) and 3020 females (46.4%). (total 6502 workers), Among the 24,249 workers younger than 55, there were 12,260 males (50.6%) and 11,989 females (49.4%).

Survey data were weighted with reference to the economically active population. The questionnaire assessed exposure to physical and chemical agents, including vibration, noise, high and low temperatures, fumes and dust, solvents, skin contact with chemicals, and passive smoking. The methodology and questionnaire were almost identical to those of the European Working Conditions Survey [10]. We defined exposure as “exposure to hazards during at least a half of the work time” and a work-related health problem as any of 13 conditions, including hearing loss, skin problems, muscle pain, depression, sleeping disturbance, etc., that may be work-related. After explanation of the survey, all participants provided written informed consent.

### Statistical analysis

Differences between aged workers (total) and those younger than 55 (total) in working conditions, exposure to hazards, and work-related health problems were analyzed using a chi-squared test. STATA (ver. 12) was used for all statistical analyses, and a *p* value below 0.05 was considered significant.

## Results

### General characteristics of study subjects

Aged workers, defined as those older than 55, worked at places with fewer employees, accounted for a smaller proportion of regular workers, received lower salaries, and accounted for a higher percentage of those employed in Elementary Occupations, relative to workers younger than 55 (Table 1). This tendency was particularly strong in workers older than 65.

**Table 1:** General characteristics of study subjects

		Age < 55	≥55 Age < 65	Age ≥ 65	*p*-value
		Frequency (%)	Frequency (%)	Frequency (%)	
Workplace size	<5 workers	5097(21.5)	1052(25.4)	647(31.2)	*P* < 0.001
	5 ~ <30 workers	9158(38.5)	1761(42.6)	1051(50.7)	
	30 ~ <50 workers	2774(11.7)	412(10.0)	165(8.0)	
	50 ~ <300 workers	4583(19.3)	660(16.0)	187(9.0)	
	≥300 workers	2149(9.0)	254(6.1)	24(1.2)	
	Total	23,761(100.0)	4139(100.0)	2074(100.0)	
Type of employment	Regular	19,041(79.0)	2528(59.2)	602(27.5)	*P* < 0.001
	Temporary	3782(15.7)	1052(24.6)	1037(47.3)	
	Daily	1280(5.3)	689(16.1)	552(25.2)	
	Total	24,103(100.0)	4269(100.0)	2191(100.0)	
Monthly salary (Korean won)	<1,000,000	1622(9.4)	544(18.2)	1040(62.0)	*P* < 0.001
	1,000,000 ~ <2,000,000	6007(35.0)	1326(44.4)	528(31.5)	
	2,000,000 ~ <3,000,000	5226(30.4)	549(18.4)	75(4.5)	
	3,000,000 ~ <4,000,000	2750(16.0)	318(10.6)	22(1.3)	
	≥4,000,000	1575(9.2)	251(8.4)	12(0.7)	
	Total	17,180(100.0)	2988(100.0)	1677(100.0)	
Major Categories of Occupation(KSCO)	Managers	466(1.9)	120(2.8)	14(0.6)	*P* < 0.001
	Professionals and Related Workers	3753(15.5)	268(6.3)	41(1.9)	
	Clerks	7321(30.3)	420(9.8)	43(2)	
	Service Workers	3353(13.9)	710(16.5)	207(9.4)	
	Sales Workers	3550(14.7)	331(7.7)	43(2)	
	Skilled Workers Related to Agriculture, Forestry and Fishery	44(0.2)	37(0.9)	51(2.3)	
	Craft and Related Trades Workers	2357(9.8)	483(11.3)	92(4.2)	
	Workers Related to Equipment, Machine Operating and assembling	1479(6.1)	366(8.5)	57(2.6)	
	Elementary Occupations	1835(7.6)	1559(36.3)	1658(75.2)	
	Total	24,158(100.0)	4294(100.0)	2206(100.0)	

Total numbers are not identical due to non-response.

### Occupations dominated by aged workers

Table 2 shows 20 occupations, in descending order of the total number of aged workers, among the 149 occupations defined as minor categories (identified by three digits) by the Korean Standard Classification of Occupations (KSCO). In 2014, there were 3,428,823 aged workers, and this accounted for 18.1% of the 18,945,403 workers in South Korea. A total of 2,439,330 of these aged workers (71.1%) were employed in the 20 listed occupations. ‘Elementary Occupations’, which the KSCO classifies as a major category (9), accounted for the largest proportion of aged workers among the 20 occupations (45%), and workers employed in the nine ‘Elementary Occupations’ accounted for 42.3% of all 3,428,823 aged workers. These ‘Elementary Occupations’ were: ‘Cleaner and Sanitation Workers’ (941), ‘Guards and Ticket Examiners’ (942), ‘Elementary Occupations Related to Food’ (952), ‘Elementary Occupations Related to Construction and Mining’ (910), ‘Domestic Chores and Infant Rearing Helpers’ (951), ‘Elementary Occupations related to Agriculture and Fishery’ (991), ‘Elementary Occupations Related to Production’ (930), ‘Elementary Occupations Related to Other Services’ (999), and ‘Deliverers’ (922). There were five occupations of skilled manual workers that accounted for 17.4% of the total aged workers: ‘Automobile Drivers’ (873), ‘Service Workers Related to Medical and Welfare’ (421), ‘Chefs and Cooks’ (441), ‘Technical Worker Related to Construction’ (772), and ‘Technical Workers Related to Construction Finishing’ (773). There were also workers in six skilled non-manual occupations that accounted for 11.4% of the total aged workers: ‘Clerks Related to Administration’ (312), ‘Store Sales Workers’ (521), ‘Sales Workers’ (510), ‘Administration Clerks’ (311), ‘Religious Professionals and Associate Professionals’ (248), and ‘Teachers’ (252).

**Table 2:** Occupations that are dominated by aged workers, (unit; number)

Occupation, Korean Standard Classification of Occupations (No. of minor category)	Workers younger than 55	Workers aged 55 or older	Total workers	Proportion of aged workers
Total	15,516,580	3,428,823	18,945,403	18.1%
Cleaner and Sanitation Workers (941)	154,789	514,734	669,523	76.9%
Guards and Ticket Examiners (942)	34,376	206,817	241,193	85.7%
Automobile Drivers (873)	354,404	174,921	529,325	33.0%
Elementary Occupations Related to Food(952)	213,327	154,840	368,167	42.1%
Clerks Related to Administration (312)	1,845,084	149,151	1,994,235	7.5%
Service Workers Related to Medical and Welfare (421)	142,735	148,189	290,924	50.9%
Elementary Occupations Related to Construction and Mining (910)	252,974	145,535	398,509	36.5%
Domestic Chores and Infant Rearing Helpers (951)	65,415	127,163	192,578	66.0%
Chefs and Cooks (441)	347,880	115,729	463,609	25.0%
Elementary Occupations related to Agriculture and Fishery (991)	38,169	102,634	140,803	72.9%
Elementary Occupations Related to Production (930)	299,138	93,173	392,311	23.7%
Technical Worker Related to Construction (772)	152,831	91,909	244,740	37.6%
Elementary Occupations Related to Other Services (999)	56,680	72,691	129,371	56.2%
Store Sales Workers (521)	841,347	66,002	907,349	7.3%
Technical Workers Related to Construction Finishing (773)	151,042	65,353	216,395	30.2%
Sales Workers (510)	471,593	62,289	533,882	11.7%
Administration Clerks (311)	362,739	41,447	404,186	10.3%
Religious Professionals and Associate Professionals (248)	88,487	38,701	127,188	30.4%
Deliverers (922)	173,066	34,361	207,427	16.6%
Teachers (252)	356,014	33,691	389,705	8.6%

Occupations in which aged workers accounted for more than 50% of the total workers were: ‘Cleaner and Sanitation Workers’ (941), ‘Guards and Ticket Examiners’ (942), ‘Service Workers Related to Medical and Welfare’ (421), ‘Domestic Chores and Infant Rearing Helpers’ (951), ‘Elementary Occupations related to Agriculture and Fishery’ (991), and ‘Elementary Occupations Related to Other Services’ (999).

### Exposure of aged workers to hazards in different occupations

Aged workers classified as ‘Technical Workers Related to Construction’ (772) reported the greatest exposure to vibration (61.3%), followed by ‘Elementary Occupations Related to Construction and Mining’ (910) (47.8%) (Table 3). Exposure to noise was also greatest for aged workers classified as ‘Technical Workers Related to Construction’ (772) reported the greatest exposure to vibration (45.1%), followed by ‘Elementary Occupations Related to Construction and Mining’ (910) (40.2%). Exposure to high temperatures was reported by aged workers who had outdoor jobs, such as ‘Elementary Occupations Related to Construction and Mining’ (910) (53.7%), ‘Technical Workers Related to Construction’ (772) (48.6%), ‘Technical Workers Related to Construction Finishing’ (773) (46.0%), and ‘Elementary Occupations related to Agriculture and Fishery’ (991) (40%); exposure to low temperatures was most common in the same occupations. Exposure to fumes and dust was most common in ‘Technical Workers Related to Construction’ (51.4%), followed by ‘Elementary Occupations Related to Construction and Mining’ and ‘Technical Workers Related to Construction Finishing’. Exposure to solvents was most common in ‘Technical Workers Related to Construction Finishing’ (31.0%), followed by ‘Technical Workers Related to Construction’. Skin contact with chemicals was most common in ‘Technical Workers Related to Construction Finishing’, and then ‘Elementary Occupations Related to Production’. Exposure to passive tobacco smoke was most common in ‘Technical Workers Related to Construction Finishing’, and then ‘Technical Workers Related to Construction’.

**Table 3:** Proportion of aged workers exposed to hazards in different occupations (%)

Occupation, Korean Standard Classification of Occupations (No. of minor category)	Vibration	Noise	High temperature	Low temperature	Fumes and dust	Solvents	Skin contact with chemicals	Passive smoking	Tiring or painful positions	Lifting or moving people	Carrying or moving heavy loads	Standing long	Repetitive movements
Cleaner and Sanitation Workers (941)	6.1	4.4	28.3	15.0	17.9	2.1	4.0	3.9	43.9	3.4	20.8	66.1	68.1
Guards and Ticket Examiners (942)	1.8	2.5	13.9	8.7	4.8	1.0	1.3	3.0	18.3	4.4	12.2	28.1	19.9
Automobile Drivers (873)	32.0	11.5	12.7	8.2	22.6	3.0	1.9	5.9	53.7	7.8	14.5	13.5	70.6
Elementary Occupations Related to Food(952)	6.1	7.9	21.2	7.3	3.7	1.2	2.4	2.4	46.7	5.5	23.6	72.4	67.9
Clerks Related to Administration (312)	10.5	6.1	6.1	3.4	4.4	1.7	1.4	1.0	13.6	4.4	7.8	15.0	30.4
Service Workers Related to Medical and Welfare (421)	4.0	1.8	7.1	4.9	2.2	0.5	0.9	0.9	30.9	42.0	15.6	30.8	40.5
Elementary Occupations Related to Construction and Mining (910)	47.8	40.2	53.7	25.5	50.2	8.5	6.4	18.3	67.9	8.5	72.2	83.8	69.8
Domestic Chores and Infant Rearing Helpers (951)	1.7	2.2	5.6	5.0	1.1	1.7	1.7	1.1	34.8	32.6	8.4	21.7	35.6
Chefs and Cooks (441)	8.3	9.2	30.7	8.9	7.6	1.0	1.6	1.6	47.5	7.0	24.1	84.2	71.2
Elementary Occupations related to Agriculture and Fishery (991)	6.9	4.6	40.0	13.1	14.6	1.5	2.3	0.8	66.4	3.8	29.0	41.9	78.5
Elementary Occupations Related to Production (930)	36.4	25.6	25.6	10.1	21.7	7.8	7.0	3.1	56.6	6.3	32.0	50.4	85.2
Technical Worker Related to Construction (772)	61.3	45.1	48.6	23.2	51.4	10.6	5.7	24.1	65.3	6.3	58.2	88.0	83.8
Elementary Occupations Related to Other Services (999)	7.9	7.9	11.1	7.9	12.7	0.0	0.0	6.4	30.7	1.6	7.9	65.1	49.2
Store Sales Workers (521)	3.8	6.9	8.8	9.3	5.6	1.3	0.6	1.9	42.6	7.5	24.2	71.6	55.9
Technical Workers Related to Construction Finishing (773)	42.0	36.0	46.0	22.0	50.0	31.0	28.3	27.0	61.0	14.0	52.0	70.0	76.0
Sales Workers (510)	2.1	2.0	4.8	3.4	0.7	0.7	0.0	2.0	9.6	0.0	4.1	21.2	21.9
Administration Clerks (311)	4.0	4.0	9.1	2.6	2.6	0.0	0.0	0.0	15.8	2.6	6.6	13.2	38.2
Religious Professionals and Associate Professionals (248)	0.0	0.0	0.0	0.0	0.0	0.0	0.0	0.0	9.1	0.0	4.6	36.4	27.3
Deliverers (922)	12.3	7.0	26.3	10.5	7.0	0.0	0.0	3.5	38.6	0.0	63.2	56.1	52.6
Teachers (252)	8.2	8.2	9.8	4.9	9.8	0.0	1.6	1.6	19.7	5.0	3.3	88.5	57.4
Workers 55 or older (total)	17.3	13.0	22.9	11.6	16.0	3.6	3.8	5.0	38.4	7.4	23.4	50.7	55.9
Workers younger than 55 (total)	14.6	11.0	11.5	6.9	8.8	2.7	2.9	3.3	27.9	5.1	15.7	41.9	49.9
*P*-value#	*P* < 0.001	*P* < 0.001	*P* < 0.001	*P* < 0.001	*P* < 0.001	*P* < 0.001	*P* < 0.001	*P* < 0.001	*P* < 0.001	*P* < 0.001	*P* < 0.001	*P* < 0.001	*P* < 0.001

#Differences between aged workers (total) and those younger than 55 (total) using a chi-squared test

Exposure to tiring or painful positions was most common in ‘Elementary Occupations Related to Construction and Mining’ (67.9%), followed by ‘Elementary Occupations related to Agriculture and Fishery’ (991) (66.4%), ‘Technical Workers Related to Construction’ (65.3%), and ‘Technical Workers Related to Construction Finishing’. Exposure to lifting or moving people was most common in ‘Service Workers Related to Medical and Welfare’ (42%), and ‘Domestic Chores and Infant Rearing Helpers’ (32.6%). Carrying or moving heavy loads was most common in ‘Elementary Occupations Related to Construction and Mining’ (72.2%), and then ‘Deliverers’ (63.2%), ‘Technical Workers Related to Construction’ (58.2%), and ‘Technical Workers Related to Construction Finishing’ (52.0%). Exposure to prolonged standing was most common in ‘Teachers’, ‘Technical Workers Related to Construction ’, ‘Chefs and Cooks’, and ‘Elementary Occupations Related to Construction and Mining’. Exposure to repetitive movements was most common in ‘Elementary Occupations Related to Production’ (85.2%), ‘Technical Workers Related to Construction’ (83.8%), and ‘Elementary Occupations related to Agriculture and Fishery’ (78.5%). Furthermore, aged workers (total) were more likely to report all of these hazards than young workers (total).

These results showed that aged workers in many occupations reported frequent exposure to ergonomic hazards such as tiring or painful positions, carrying or moving heavy loads, and repetitive movements. These aged workers also reported frequent exposure to physical factors such as noise, vibration, high and low temperatures, and chemical factors, such as solvents and other chemicals, in different specific occupations.

### Work-related health problems of aged workers in different occupations

Hearing loss and skin problems were most common in ‘Technical Workers Related to Construction’ and ‘Elementary Occupations Related to Production’ (2.8 and 3.1%, respectively) (Table 4). Back pain was most common in ‘Elementary Occupations related to Agriculture and Fishery’ (37.9%), and was reported about 20%, in most occupations. Upper extremity pain, and lower extremity pain were reported in most occupations, and the frequencies were about 40 ~ 50% and 30 ~ 40%, respectively. Furthermore, aged workers (total) were more likely to report musculoskeletal symptoms than young workers (total). Headache and eye fatigue were most common in ‘Automobile Drivers’ and ‘Elementary Occupations Related to Production’, each with a frequency of about 20%. Aged workers (total) were less likely to report headache and eye fatigue than young workers (total). Abdominal pain and breathing difficulty were most common in ‘Elementary Occupations related to Agriculture and Fishery’. Cardiovascular disease was the most common disease among ‘Religious Professionals and Associate Professionals’. Injury due to accidents was most common in machinery-handling occupations, including ‘Technical Workers Related to Construction’, ‘Technical Workers Related to Construction Finishing’, ‘Elementary Occupations Related to Construction and Mining’, and ‘Elementary Occupations Related to Production’. Injury due to accidents was also common in ‘Sales Workers’ and ‘Religious Professionals and Associate Professionals’. Furthermore, aged workers (total) were more likely to report occupational injuries than young workers (total). Depression and anxiety disorder was most common among ‘Domestic Chores and Infant Rearing Helpers’ (1.7%), followed by other occupations that are dominated by women. General fatigue was present in about 20 ~ 30% of aged workers in most occupations, and aged workers (total) were more likely to report this symptom than young workers (total). Sleep disturbance was most common in ‘Elementary Occupations related to Agriculture and Fishery’ (4.5%), and ‘Religious Professionals and Associate Professionals’ (248) (4.5%) followed by ‘Guards and Ticket Examiners’ (4.0%), ‘Sales Workers’, ‘Technical Workers Related to Construction Finishing’, ‘Service Workers Related to Medical and Welfare’, ‘Domestic Chores and Infant Rearing Helpers’, and ‘Chefs and Cooks’, each with a frequency of 2 ~ 3%.

**Table 4:** Proportion of aged workers having work-related health problems in different occupations. (%)

Occupation, the Korean Standard Classification of Occupations (No. of minor category)	Hearing loss	Skin problems	Back pain	Upper extremity pain	Lower extremity pain	Headache and eye fatigue	Abdominal pain	Breathing difficulty	Cardiovas-cular disease	Injury due to accident	Depression and anxiety disorder	General fatigue	Sleep disturbance
Cleaner and Sanitation Workers (941)	0.9	1.1	21.2	38.6	33.3	7.9	0.6	0.2	0.7	0.3	0.4	20.7	0.6
Guards and Ticket Examiners (942)	0.5	0.5	7.6	20.4	16.5	9.9	0.5	0.2	0.5	0.5	0.5	20.2	4.0
Automobile Drivers (873)	1.5	1.8	17.7	44.1	32.7	24.6	0.7	0.7	0.0	0.4	0.7	22.8	1.8
Elementary Occupations Related to Food (952)	0.0	0.6	17.6	47.3	42.4	6.7	0.0	0.6	1.2	2.4	0.0	27.3	0.0
Clerks Related to Administration (312)	0.0	0.0	3.0	13.1	5.4	15.2	0.0	0.0	0.3	0.0	0.0	32.7	1.7
Service Workers Related to Medical and Welfare (421)	0.4	0.8	21.7	41.5	28.5	7.9	0.0	0.0	0.4	1.6	1.2	29.6	2.4
Elementary Occupations Related to Construction and Mining (910)	1.7	1.0	22.9	58.9	49.2	9.1	0.7	0.7	0.3	4.0	1.0	31.7	0.7
Domestic Chores and Infant Rearing Helpers (951)	0.6	0.6	27.5	41.8	27.5	6.0	1.1	0.0	0.0	0.6	1.7	26.9	2.2
Chefs and Cooks (441)	0.3	0.6	23.7	51.3	40.5	10.4	0.3	0.0	1.3	2.2	0.3	29.8	2.2
Elementary Occupations related to Agriculture and Fishery (991)	1.5	2.3	37.9	53.0	46.2	14.4	2.3	3.0	0.8	3.0	0.0	28.8	4.5
Elementary Occupations Related to Production (930)	1.6	3.1	25.6	51.9	38.8	22.5	0.0	0.8	2.3	4.7	0.0	27.9	0.8
Technical Worker Related to Construction (772)	2.8	2.8	26.1	61.3	44.4	12.7	0.0	0.0	0.0	3.5	0.7	32.4	0.0
Elementary Occupations Related to Other Services (999)	0.0	0.0	7.9	19.0	14.3	12.7	0.0	0.0	0.0	0.0	0.0	17.5	0.0
Store Sales Workers (521)	0.0	0.6	17.9	28.4	32.7	13.0	0.0	0.0	0.0	0.6	0.0	25.9	0.6
Technical Workers Related to Construction Finishing (773)	1.0	2.0	30.7	54.5	42.6	7.9	0.0	2.0	0.0	5.9	0.0	35.6	3.0
Sales Workers (510)	0.0	0.0	2.0	15.6	8.8	18.4	1.4	0.0	0.0	4.1	1.4	21.1	3.4
Administration Clerks (311)	0.0	1.3	7.8	11.7	9.1	16.9	1.3	0.0	0.0	0.0	1.3	10.4	1.3
Religious Professionals and Associate Professionals (248)	0.0	0.0	9.1	9.1	9.1	18.2	0.0	0.0	4.5	4.5	0.0	4.5	4.5
Deliverers (922)	0.0	0.0	14.0	49.1	36.8	14.0	0.0	0.0	1.8	0.0	0.0	38.6	0.0
Teachers (252)	0.0	0.0	11.5	19.7	16.4	11.5	1.6	1.6	0.0	0.0	0.0	14.8	0.0
Workers 55 or older (total)	1.2	1.0	16.7	36.2	28.2	11.6	0.5	0.4	0.6	1.4	0.6	22.7	1.6
Workers younger than 55 (total)	1.1	1.2	10.3	25.6	16.1	15.8	0.6	0.3	0.2	1.1	0.7	18.7	1.7
*p*-value#	*P* = 0.615	*P* = 0.110	*P* < 0.001	*P* < 0.001	*P* < 0.001	*P* < 0.001	*P* = 0.206	*P* = 0.667	*P* < 0.001	*P* = 0.041	*P* = 0.178	*P* < 0.001	*P* = 0.809

#Differences between aged workers (total) and those younger than 55 (total) using a chi-squared test.

## Discussion

The KSCO categorizes occupations based on the skills required to carry out the necessary tasks and duties. Skill has two dimensions: skill level and skill specialization. Skill level is a function of the complexity and range of tasks and duties to be performed. According to the International Standard Classification of Occupations (ISCO), an individual obtains a skill level by formal education, and informally by on-the job training and from previous experience in a related occupation [11]. Thus, the concept of skill level, applied to the classification of occupations, places more emphasis on the ability to perform a job than the requirement for a formal education. Bearing in mind the international character of the classification, there are four skill levels. Occupations at Skill Level 1 typically require the performance of simple and routine physical or manual tasks, and may require physical strength and/or endurance (e.g. the nine Elementary Occupations). Occupations at Skill Level 2 typically require the performance of tasks such as operating machinery and electronic equipment; driving vehicles; maintenance and repair of electrical and mechanical equipment; and manipulation, ordering, and storage of information. Almost all occupations at Skill Level 2 require the ability to read information, make written records of work completed, and accurately perform simple arithmetical calculations. Many occupations at this skill level require a high level of manual dexterity (e.g. the minor categories of 311, 312, 510, 521, 421, 441, 772, 773, and 873). Occupations at Skill Level 3 typically require the performance of complex technical and practical tasks that rely upon an extensive body of factual, technical, and procedural knowledge in a specialized field (e.g. the minor categories of 248 and 252). Occupations at Skill Level 4 typically require the performance of tasks that rely upon complex problem-solving, decision-making, and creativity, and are based on an extensive body of theoretical and factual knowledge in a specialized field (e.g. the minor categories of 248 and 252).

The 20 occupations dominated by aged workers can be classified as: *(a)* ‘Elementary Occupations’, in which aged workers can perform simple manual tasks without specific training, and *(b)* Skill Level 2 occupations, in which aged workers can continue to work due to the skills they acquired during their long time of employment. Very few aged workers were in occupations at Skill Levels 3 and 4.

Workers in the nine ‘Elementary Occupations’ accounted for 42.3% of all aged workers, and 59.5% of aged workers were employed in the 20 occupations analyzed here. In Korea, the average retirement age from primary jobs is about 50, after which most retirees get a series of second jobs, mostly low-quality ‘Elementary Occupations’ [3]. They also tend to work at places with fewer employees, account for a smaller proportion of regular workers, and receive lower salaries than workers younger than 55 (Table 1). Aged workers employed in Elementary Occupations often perform simple but physically demanding jobs in new environments, and thus have increased risk of occupational injury. In addition, age-related musculoskeletal deterioration may increase the risk of occupational injuries in aged workers [12, 13]. Thus, aged workers are most susceptible to injury when employed in ‘Elementary Occupations’.

In occupations at Skill Level 2, the skill and experience that aged workers have acquired over time may offset their decreased performance due to aging. However, aged manual workers in occupations at Skill Level 2 are exposed to job-specific hazards. According to the present study, ‘Automobile Drivers’ are exposed to whole body vibration, compatible with previous studies [14–16], and ‘Service Workers Related to Medical and Welfare’ are exposed to lifting or moving people, in agreement with previous study [17]. The present study also showed that ‘Chefs and Cooks’ were exposed to high temperatures and long standing, compatible with previous studies [18, 19]. ‘Technical Workers Related to Construction’ were exposed to vibrations, noises, awkward postures, carrying heavy loads, and long standing, as reported in a previous study [20]. ‘Technical Workers Related to Construction Finishing’ were exposed to solvents.

However, workers in skilled non-manual occupations were less likely to report most occupational hazards and work-related health problems. These low-risk occupations were ‘Clerks Related to Administration’ (312), ‘Administration Clerks’ (311), ‘Store Sales Workers’ (521), ‘Sales Workers’ (510), ‘Religious Professionals and Associate Professionals’ (248), and ‘Teachers’ (252).

Taken together, these results indicate we should focus on ‘Elementary Occupations’, and then skilled manual occupations (e.g. ‘Automobile Drivers’, ‘Service Workers Related to Medical and Welfare’, ‘Chefs and Cooks’, ‘Technical Worker Related to Construction’, and ‘Construction Finishing’) to improve the health and safety of aged workers. These target occupations employ about 60% of all aged workers, have greater proportions of aged workers, and have more occupational hazards.

The present study indicated that aged workers in the target occupations had more frequent exposure to ergonomic hazards, such as tiring or painful positions, carrying or moving heavy loads, and repetitive movements, and also had more work-related musculoskeletal disorders and general fatigue than the average for all aged workers

Injury due to accident was frequently reported in machinery-handling occupations, such as ‘Technical Workers Related to Construction Finishing’ and ‘Elementary Occupations Related to Production’. In particular, the frequency of injury due to accident in aged workers who were ‘Technical Workers Related to Construction Finishing’ and ‘Elementary Occupations Related to Production’ were 5.9 and 4.7%, respectively, more than three times the average of all aged workers (1.4%). The present findings are compatible with those of previous papers [5, 20, 21]. In particular, aged workers who have poor muscle strength and elasticity and limited range of motion in joints, are more likely to complain of musculoskeletal symptoms and suffer from work-related injuries than young workers [4, 22]. The higher frequency of injury due to accident in aged workers who were in ‘Sales Workers’ and ‘Religious Professionals and Associate Professionals’ may be related to traffic accidents.

The present study has several strengths. We focused on high-risk workplaces that are dominated by aged workers, rather than on the entire population of aged workers. The work ability of aged workers can be efficiently and effectively maintained and promoted only when employers take a leadership role in activities that promote their work ability, and when the aged workers themselves actively participate in these activities in high-risk workplaces. In addition, job-specific risks for aged workers can be assessed in these workplaces, and this can provide better management of job-specific occupational hazards and establishment of age-friendly workplaces.

The present study also had several limitations. First, we used self-reported data, instead of objective findings. Second, we did a cross-sectional study. Therefore, we cannot infer causality.

The present study has several practical implications. First, we identified occupations that were dominated by aged workers. These occupations, ‘Elementary Occupations’ in particular, should be the target of interventions that aim to improve the occupational safety and health of aged workers. Second, we assessed job-specific hazards and health problems in the different occupations that employ the most aged workers. This assessment provides a basis on which age-friendly occupational health and safety guidelines can be developed to improve the health and safety of aged workers.

## Conclusions

Employers should try to reduce job-specific hazards to which aged workers are exposed to prevent work-related disorders in occupations dominated by aged workers. In addition, better prevention of ergonomic risk factors and preventive measures, such as a muscle strengthening and stretching program, may help to prevent work-related musculoskeletal disorders.

## Abbreviations

ISCO: International Standard Classification of Occupations
KSCO: Korean Standard Classification of Occupations
NCD: Non-communicable diseases
